# An Information Gap in DNA Evidence Interpretation

**DOI:** 10.1371/journal.pone.0008327

**Published:** 2009-12-16

**Authors:** Mark W. Perlin, Alexander Sinelnikov

**Affiliations:** 1 Cybergenetics, Pittsburgh, Pennsylvania, United States of America; 2 Genetica, Cincinnati, Ohio, United States of America; University of East Piedmont, Italy

## Abstract

Forensic DNA evidence often contains mixtures of multiple contributors, or is present in low template amounts. The resulting data signals may appear to be relatively uninformative when interpreted using qualitative inclusion-based methods. However, these same data can yield greater identification information when interpreted by computer using quantitative data-modeling methods. This study applies both qualitative and quantitative interpretation methods to a well-characterized DNA mixture and dilution data set, and compares the inferred match information. The results show that qualitative interpretation loses identification power at low culprit DNA quantities (below 100 pg), but that quantitative methods produce useful information down into the 10 pg range. Thus there is a ten-fold *information gap* that separates the qualitative and quantitative DNA mixture interpretation approaches. With low quantities of culprit DNA (10 pg to 100 pg), computer-based quantitative interpretation provides greater match sensitivity.

## Introduction

DNA identification is a powerful forensic tool for solving and preventing crime [1]. However, DNA evidence is collected from the field under real world conditions, and may produce less pristine data than a reference specimen obtained from an individual in a controlled setting. Two common sources of data ambiguity in biological evidence are (a) DNA mixtures from multiple contributors, and (b) low template DNA (LT-DNA) that is under 100 pg in the evidence sample.

DNA mixtures can be highly probative evidence in a sexual assault crime (e.g., stranger rape), where a culprit's semen mixes with a victim's epithelial cells [2]. Mixtures of culprit and victim in other violent crimes (e.g., homicide) can help establish that a suspect was involved in a criminal event. Property crime DNA evidence [3] is often mixed, low template, or both. A low amount of DNA template (in any type of crime) produces less amplified signal, creating ambiguous data whose forensic interpretation may yield less identification information [4].

These DNA challenges have a major impact on crime laboratory practice. Difficult samples may consume inordinate examiner time and produce suboptimal information, generating DNA backlogs and inconclusive results [5]. Yet such challenging evidence may be extremely important in protecting the public from dangerous criminals. One laboratory estimated that timely DNA examination of all property crimes and sexual assaults would prevent 100,000 stranger rapes in the United States [6]. This is partly because burglary and rape are both crimes of opportunity perpetrated by similarly specialized career criminals [7], so incarcerating burglars can help prevent rapes.

DNA data are generated through a linear amplification and readout process in which quantitative allele events are combined arithmetically. Such linearly generated DNA data can be mathematically described through a quantitative linear model [8], [9]. Some practitioners do analyze mixtures using quantitative peak information [10]. However, most forensic DNA interpretation currently uses instead a qualitative Boolean logic of all-or-none allele events [11].

There is little consensus on the interpretation of LT-DNA and mixtures. Qualitative methods begin by applying a peak height threshold to the quantitative DNA signal to retain or discard data peaks, removing peak height information. The current controversy questions the choice of numerical threshold value (ranging from 50 to 300 units), and how many thresholds to apply (one [12], two [13] or many [14]). Practitioners debate whether mixture interpretation should account for known contributors [15], [16], or instead ignore victim genotypes [13], [17]. Some scientists propose how to interpret LT-DNA [4], while others decry the practice altogether [18]. It has been recognized [19] that mathematically modeling the quantitative data [8], [20] could resolve these “threshold” issues, and there has been considerable progress in statistical computer models for interpreting complex DNA evidence [9], [21], [22], [23].

This ongoing debate raises some important questions. What are the true limits of DNA interpretation for mixtures and low template samples? What available interpretation methods can extract the most DNA information for identifying criminals? How do quantitative DNA mixture interpretation approaches compare with current qualitative practice? Understanding these issues can help society allocate effective crime fighting DNA resources for increasing public safety.

In this paper, we examine the information extracted by quantitative and qualitative DNA interpretation methods. We apply both methods to the same mixture data set of varying contributor weights and DNA quantities. We identify an *information gap* between the two approaches: qualitative methods are limited to culprit DNA quantities above 100 pg, whereas quantitative methods can extend meaningful interpretation down to 10 pg. We show how analyzing the information gap was helpful in presenting DNA evidence in court. We conclude that quantitative methods may be preferable when interpreting LT-DNA mixture evidence.

The overall aim of the study was to compare the relative efficacy of newer quantitative computer-based methods of DNA mixture interpretation to current qualitative manual methods. We did this by measuring the sensitivity of each method, exploiting a new observation that a linear relationship exists between the (logarithm of) DNA quantity and DNA match information. We observed that quantitative mixture interpretation extends the current detection limits of qualitative methods by an order of magnitude, thereby achieving the aim of the study.

## Methods

We examine alternative approaches to DNA mixture interpretation. We first present a quantitative linear model for understanding the generation of mixed and low template STR data. We explain how the probability model accounts for stochastic effects. We then show how computer implementation of this quantitative model can infer genotypes for the contributors to the data. We also describe the current qualitative mixture interpretation methods used in crime laboratories. We use an information measure based on genotype match rarity that can be used to compare these quantitative and qualitative inference methods. We also show how standard DNA match statistics can be derived from this information measure. For objectivity [24], we always first infer a genotype (committing to an answer at all loci), and only afterwards in a second step match it against another genotype [25]. We also describe the data design, software and parameters used in this study.

### Mixture Data Model

In short tandem repeat (STR) genotyping, alleles correspond to the length of an amplified polymerase chain reaction (PCR) product, which is assayed by size separation on a DNA sequencer [26], [27]. A nanogram of DNA from a single individual produces one or two tall allele peaks, along with smaller artifact peaks. A DNA mixture, though, has multiple contributors and may produce a more complex data pattern [20], [28]. Lower DNA amounts reduce the observed peak heights and increase stochastic effects. In STR analysis, both PCR amplification and sequencer detection are fundamentally linear processes, so a mixture of genotypes produces a signal that is approximately the sum of the separate genotype signals [29].

We can model the quantitative data at STR locus 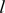 (of 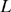 loci) using several variables. Data vector 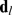 forms a pattern that maps DNA product lengths into their observed quantitative peak heights (or areas). With 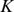 contributors to the data, we represent the 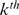 contributor genotype parameter at locus 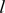 as a vector 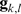, where the DNA length entries contain allele counts that sum to 1 [8]. A heterozygote genotype vector 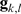 contains two 0.5 entries, while a homozygote has a single 1 entry; all other vector entries are 0 [30]. The mixture weight parameter is represented as a vector 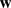 whose components sum to 1 (i.e., 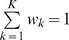). The total DNA quantity at locus 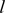 is given by the mass parameter 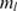. With these three variables, a quantitative linear model of data pattern 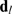 at locus 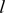 has an expected vector value 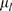 given by the weighted genotype sum in equation (1).
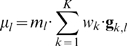
(1)A more complete model would also address PCR stutter, relative amplification, degraded DNA, dye separation and background noise [31]. A useful hierarchical refinement models mixture weight individually at every locus, with each weight drawn from a common DNA template mixture distribution [32].

There is random variation in the observed peak heights resulting from PCR amplification and sequencer detection. PCR is a branching process [33] where the random element comes from DNA replication efficiency, modeled by a copy (or not) Bernoulli event for each DNA molecule at every cycle [34]. Computer simulations [35] in this Bernoulli model show that the amplification variance scales with the peak height *y*, an estimate of DNA quantity. Empirical studies demonstrate that PCR follows a stochastic Poisson count distribution, where the product variance is proportional to DNA quantity [36]. As with other event count models, it is useful to add a dispersion factor 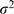 to account for model deviation [37], so we model the amplification variance of a peak as 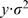. Sequencer detection variation is independent of the DNA quantity, and can be modeled separately by a constant variance 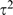 parameter. We also note that the data peaks should be independent of one another.

With these considerations in mind, we write the data covariance matrix 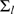 as in equation
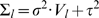
(2)where 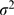 is the amplification dispersion, 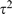 is the detection variation, and 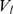 is a diagonal matrix 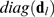 of peak heights. We can then linearly model the data vector 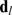 using a truncated (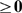) multivariate normal distribution 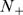 of the mean vector 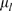 and covariance matrix 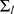 [8] as in equation (3).
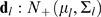
(3)Other square deviation data models can be used [38], [39], as well as nonnormal distributions [40].

**Figure 1:**
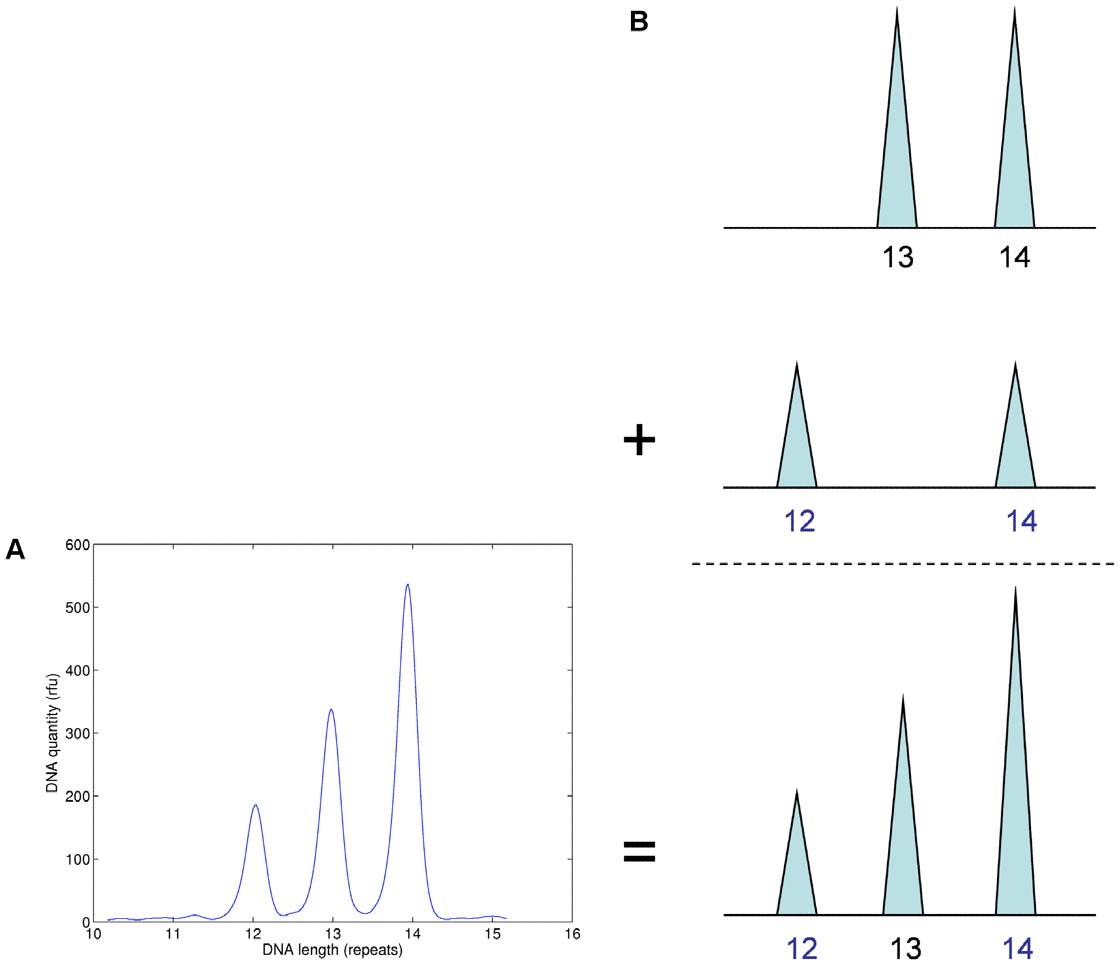
STR data can be modeled by linear superposition of genotype patterns. (a) The DNA sequencer data signal shows the Penta D STR locus for a 0.25 ng 30% culprit DNA sample C3. (b) Linearly combining genotype values 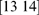 and 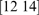 in respective 70% and 30% proportions forms a model of the observed allele peak height pattern.

We show an example data signal (Figure 1a) from the Penta D locus of sample C3, described below in the Data section. There are three alleles in the overlapping allele pairs of two contributor genotypes 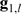 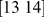 and 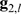 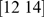. The mixture weight 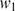 of the first contributor “A” is 70%, and the weight 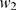 of the second contributor “G” is 30%. The weighted sum of the genotype vectors forms an ascending peak pattern (Figure 1b). The total allelic peak mass 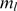 is 1,062 relative fluorescent units (rfu). Visually, we see a good fit between the quantitative peak height data pattern 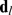 and the quantitative linear estimate of equation (1).

### Quantitative Genotype Inference

Suppose that 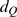 is a questioned evidence sample. To make comparisons with other genotypes, we want to infer a contributor genotype Q for sample 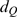. We are particularly interested in situations where there is uncertainty in Q. Therefore, our task is to infer genotype Q's posterior probability mass function (pmf) 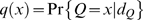, say, for contributor 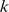 at locus 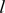 [21], [22]. Allele pair 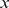 belongs to a finite set X of genotype values.

To infer the pmf 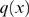 of genotype 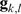, we use the joint probability distribution over all the relevant random variables [41]. The likelihood function was given by equation (3). The prior probability assignments are given in equations (4).
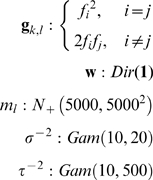
(4)The genotype prior probability 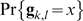 at allele pair 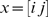 is a product of population allele frequencies 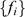. The mixture weight 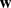 has a uniform prior distribution over the 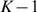 dimensional simplex for the 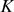 contributors. The locus mass 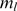 prior is a (nonnegative) truncated normal distribution on feasible total peak rfu values. The data variation parameters 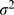 and 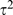 have inverse gamma prior probability distributions.

The joint probability distribution is fully specified as the product of the likelihood (3) and prior (4) distributions [42], [43]. Therefore, we can iteratively draw from the posterior probability distributions of variables 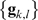, 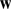, 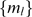, 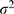 and 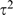 using Markov chain Monte Carlo (MCMC) computer methods [21], [32]. After progressing beyond the initial burn in cycles, the chain then samples from the joint posterior probability distribution [44]. By marginalizing to just the one genotype random variable 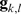 for contributor 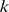 at locus 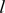, we obtain the desired probability function 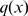 for genotype Q.

**Figure 2:**
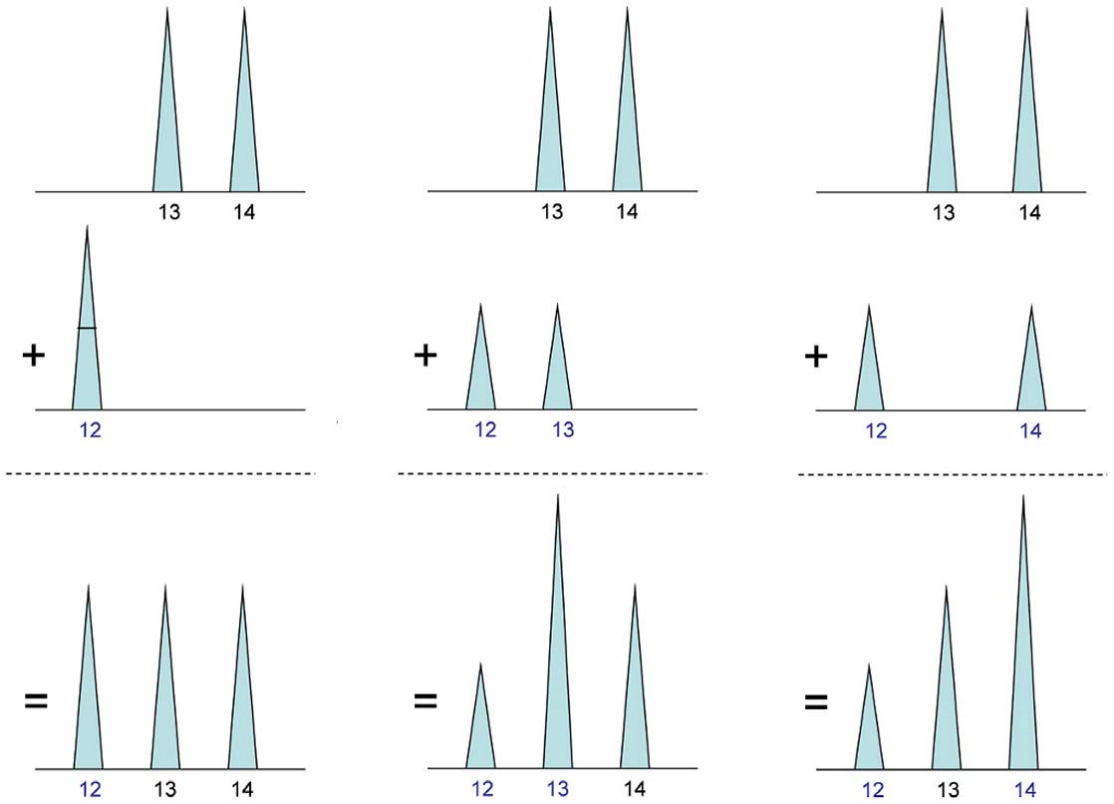
Different genotype combinations produce patterns that are compared with data to obtain a genotype probability. Linear combinations of known victim contributor 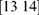 with three different unknown contributor allele pair candidates are shown at STR locus Penta D. The victim contribution is known to be 70%, and the culprit's is 30%. The allelic peak height pattern that best fits the observed data (Figure 1a) corresponds to the 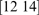 candidate (rightmost column). The other two candidates produce patterns that have a very poor fit to the quantitative data peaks. Therefore, based on a multivariate normal likelihood function, allele pair candidate 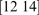 would have the greatest probability of arising from the culprit genotype.

**Figure 3:**
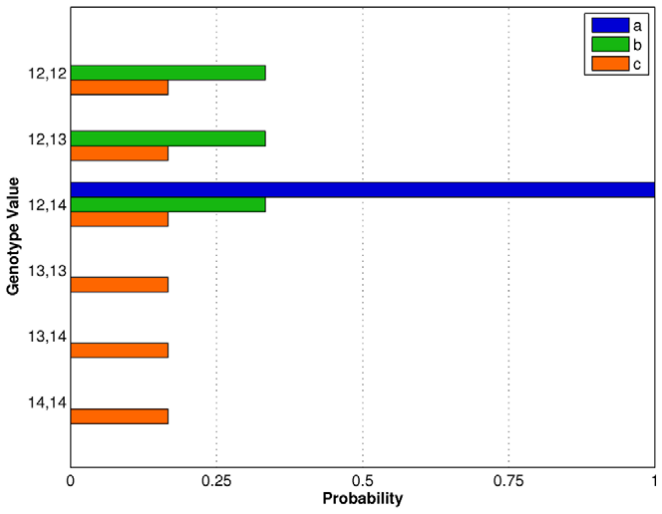
Some genotype probability distributions are more informative than others. The allele pair probabilities for six possible genotype candidates are shown for three different DNA mixture interpretation methods: (a) quantitative data modeling based on peak heights; (b) qualitative listing of genotype possibilities, filtered by consideration of an obligate culprit allele; and (c) unfiltered qualitative genotype listing that includes all allele pairs, based on “allele peaks” over threshold. A higher probability for the correct genotype value leads to greater match information.

Let us return to our example quantitative mixture data, with the three observed alleles 12, 13 and 14. Assume (as the computer might during an MCMC cycle) that the mixture weight is 70% and 30% for the two contributors, respectively, and that the first genotype (known victim) is 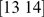. Since allele 12 should then be in the second genotype (unknown culprit), there would be three feasible allele pairings for the unknown second contributor (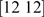, 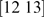, 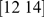). We show these three model patterns 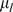 computed from equation (1), based on each corresponding feasible allele pair (Figure 2). Only one of these candidate patterns reasonably fits the quantitative data observed in Figure 1. Therefore, accounting for data peak variance and other parameters, the computer might assign the 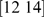 allele pair corresponding to that ascending peak pattern a probability of one, and all other genotype values a probability of zero (Figure 3, blue bar a).

### Stochastic Effects

PCR is a stochastic process [34] that yields an uncertain amount of amplified product [36]. Allele dropout can occur when either a visible allele peak falls below a qualitative interpretation threshold, or the allele does not amplify at all [31]. Probability modeling of quantitative peak data as described above can account for both situations.

Suppose that an allele produces a small amount of amplified product, with a correspondingly low peak height. Applying qualitative review, if that height were under some interpretation threshold, the peak would “drop out” from the analysis and be lost to genotype inference. This drop out does not happen when using a quantitative probability model, however. Instead, the data variance model equations (2) and (4) enter into the likelihood comparison (3) of the quantitative data pattern with any proposed genotype combination (1) in forming a genotype probability. Since the amplification variance component 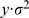 scales with the peak height *y*, the variance reflects the greater data uncertainty (viewed as a coefficient of variation) when *y* is small. Moreover, when the observed data pattern does not fit any genotype model particularly well, the dispersion factor 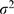 increases, further increasing modeled data uncertainty.

Now suppose that no detectable amplification occurs, so that an allele has no peak at all (e.g., its rfu is zero). Here, the 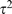 detection variation component of equation (2) comes into play. With low data, peaks have small heights, and so their variances 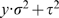 are comparable to the 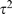 variance of missing alleles that have dropped out. When considering these peak variance values, the likelihood comparison (3) can assign genotype candidates a non-negligible probability value, even when their alleles show no peaks in the data.

In this way, quantitative STR data can convey their uncertainty via the data variance into a genotype. Greater genotype uncertainty is represented by a more diffuse probability distribution. And, as we shall presently see, a less certain genotype pmf generally reduces match LR information.

### Qualitative Genotype Inference

Qualitative genotype inference does not make full use of the quantitative peak height pattern data. Rather, qualitative review entails the sequential processing of several lists.

- **Peaks.** DNA sequencer software first analyzes an STR signal to form an initial list of quantitative data peaks. An analyst removes questionable peaks that show data artifacts (e.g., peak morphology, spikes, elevated baseline), producing a final peak list.
- **Alleles.** To form an initial allele list, an analyst applies a fixed, preset peak threshold to the peak list, discarding all peaks whose heights are below this threshold [45]. After accounting for allele artifacts (e.g., stutter position, dye bleedthrough), the acceptable alleles remain on the final list. The peak thresholding process discards quantitative peak height data, creating qualitative all-or-none allele events [13].
- **Genotypes.** All possible pairs are constructed from the allele list, building an initial list of feasible genotype candidates. (This listing may be implicitly formed by match statistic software.) Some laboratories filter this list based on known genotype or peak height information to create a smaller final list of genotype values [10].

Qualitative genotype inference considers the allele pairs in the final list to be equally likely [11]. That is, with N listed allele pairs, the probability of each one is 1/N. A smaller list has a smaller N, hence a more definite belief (i.e., a larger probability 1/N) in each listed allele pair. Including more genotype values on the list broadens the probability distribution, indicating less confidence in any one particular choice.

**Figure 4:**
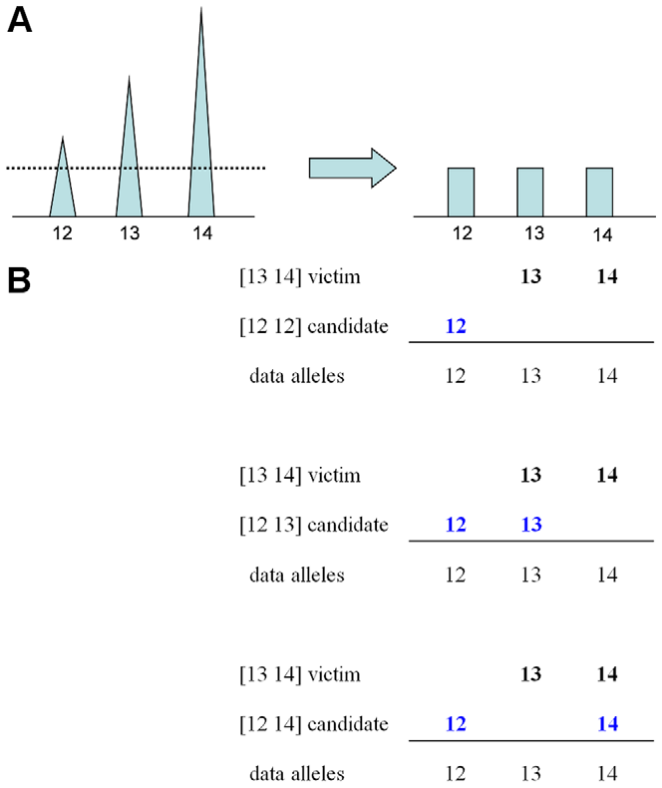
Qualitative genotype inference uses thresholds to discard data and produce a uniform genotype probability distribution. In qualitative genotype interpretation, a predetermined threshold is applied to the peak height data, retaining all peaks whose heights exceed the threshold, and discarding all other peaks. (a) This threshold operation transforms the quantitative peak height pattern into a qualitative all-or-none set of threshold-inferred alleles. (b) This data allele set can then be compared with victim (black) and candidate culprit (blue) genotype values in a match operation based on qualitative set inclusion. When accounting for the victim's genotype, all possible culprit allele pairs that combine with the victim's alleles to reproduce the data alleles are assigned equal positive probability.

To illustrate qualitative interpretation, apply a threshold of 100 rfu to our example quantitative mixture data, to obtain three alleles, 12, 13 and 14 (Figure 4a). These alleles are now all-or-none events, without quantitative peak height information and combined using Boolean logic. (For example, thresholded alleles cannot be arithmetically added together using mixture weights.) Since the known victim genotype is 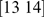, “obligate” allele 12 must appear in the culprit genotype. The three feasible genotype candidates for the unknown second contributor (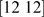, 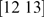, 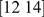) can logically combine with the 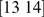 victim genotype to reproduce the observed data alleles 12, 13 and 14 (Figure 4b). Since all three candidates (in combination with the victim genotype) produce the identical Boolean allele pattern, they share the same probability of 1/3 (Figure 3, green bar b). Alternatively, a different interpretation method that did not apply the victim genotype information to the unfiltered allele pairs would infer all six genotype values, each having probability 1/6 (Figure 3, orange bar c).

### Genotype Match Information

The likelihood ratio (LR) is the cornerstone of statistical forensic inference [15]. The LR is a ratio of the probabilities of observing evidence under two alternative hypotheses [46]. The odds form of Bayes theorem [47] tells us that the posterior odds of a hypothesis relative to its alternative is the LR times the prior odds before observing evidence. Thus the LR is based solely on an evaluation of the scientific evidence, uninfluenced by prior beliefs. Evidence that favors the hypothesis has a LR>1, while unfavorable evidence has a LR<1. Multiple independent sources of evidence can be combined by multiplying their LRs.

Having inferred a probability distribution 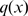 for questioned genotype Q, the next step [25] is to quantify its identification information by using a LR [48] that measures match rarity [31]. Suppose that we compare inferred genotype Q with a suspect genotype S to obtain the specific genotype match probability 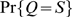. We can normalize with respect to a match probability 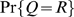 between genotype Q and a random genotype R drawn from someone other than the suspect. The ratio of the specific to random match probabilities forms the match likelihood ratio (MLR) [48] shown in equation (5).
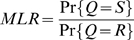
(5)


The MLR is a LR that assesses the hypothesis that the suspect contributed to the DNA evidence [48]. The MLR can be calculated directly from the genotype pmfs 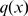, 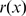 and 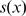 of the respective genotypes Q, R and S. When genotype Q is inferred independently of the suspect S, the MLR becomes a ratio of a sum of probability products [48], as shown in equation (6). This probability function formulation of the standard match LR facilitates calculation with uncertain genotypes.
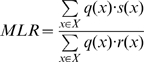
(6)


### DNA Match Statistics

Each qualitative genotype inference method produces a specific pmf 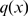 for Q. When this probability function is inserted into MLR formula (6), it reproduces a corresponding qualitative DNA match LR statistic [48]. At a mixture locus, qualitatively inferred genotype Q is a list of N allele pairs that are assigned equal probabilities. Therefore, Q's pmf has a uniform distribution
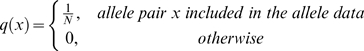
These included allele pairs form a list G of included genotype values.

Suppose that there is a matching genotype allele pair 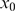 at which suspect genotype S has mass probability 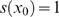 (hence is zero at other values), and that 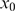 appears in Q's inclusion list G. Substituting these probability functions 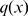 and 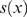 into MLR equation (6), we obtain one over the sum of population frequencies for the included genotype values, as derived in equation (7).
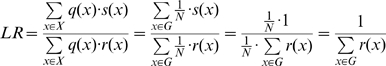
(7)


The initial qualitative genotype list comprises all possible allele pairs, and so the MLR becomes the standard “Probability of Inclusion” DNA match statistic [2]. When a victim genotype is considered, its influence may filter this genotype list into a smaller one that assigns larger probabilities to each candidate. This final genotype list specifies a 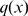 probability function from which MLR constructs a (conventionally termed) “Likelihood Ratio” DNA match statistic [16].

With autosomal (or other independently segregating) STR loci, the joint LR combines the individual locus LR results by multiplying them together [1]. The resulting Combined Probability of Inclusion (CPI) (also referred to as Random Man Not Excluded (RMNE)) and Combined Likelihood Ratio (CLR) DNA match statistics are widely used in the United States for reporting mixture results [11], [13], and are routinely calculated in the Popstats program of the FBI's CODIS database software [49]. We use CPI and CLR here as LR measures of match information for assessing qualitative genotype inference, with respect to a suspect and a population. Therefore, we must include all loci (both matching and nonmatching) in order to obtain a valid total LR statistic [50]. (Note that some forensic labs determine CPI and CLR differently, and include only matching loci in their reported statistic.)

With quantitative genotype inference, the pmf 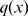 for genotype Q need not be uniform, and can take on different probabilities at different genotype values. When the victim genotype is considered as a known reference in the mixture interpretation, the resulting Q genotype is based on inferring just one genotype, that of the unknown culprit. Substituting Q's pmf 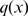 into MLR formula (6), we obtain a LR that we call “LR1” for assessing one unknown genotype. In many DNA evidence situations, no reference genotype is available. Then two unknown questioned genotypes Q must be inferred from the mixture data, and each one can be compared with the suspect genotype S by substituting its 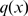 into MLR formula (6). We call the resulting likelihood ratio “LR2”, since it assesses a two unknown genotype inference.

When working with the logarithm of the LR, the joint log(LR) is the sum of the component locus log(LR)'s. The biological sciences conventionally use the common (base 10) logarithm, i.e., a number's “order of magnitude”. The common logarithm log_10_(LR) is a standard additive measure of information called the “weight of evidence” [50]. Juries tend to find DNA evidence highly persuasive once the total LR reaches a million-to-one [51], so we shall use a weight of evidence of 6 (i.e., log_10_(10^6^)) as the threshold for evidentially useful match information.

### Comparing Interpretation Methods

The strength of a mixture interpretation method resides in the DNA match score it produces. This single number supplies the weight of DNA evidence for investigative, evidentiary and exculpatory purposes. Relative to a particular suspect and reference population, the match statistic is entirely determined by the (possibly implicit) genotype pmf inferred by an analyst from the evidence data [48]. More informative methods obtain higher match scores by inferring more informative genotypes.

Our study compares the efficacy of DNA interpretation methods through the match information they each derive when applied to identical data. Every match statistic used here can be viewed as a LR:

- CPI is formally a LR, as previously observed [48, Illustrated examples, Mixtures] and summarized in equation (7) and its succeeding paragraph. We provide a detailed derivation (Appendix S1) that starts from the PI definition, introduces appropriate likelihood and prior probability functions for genotype inference, and uses MLR to prove that CPI is a LR for the standard identification hypothesis that the suspect's DNA is in the evidence.
- CLR is known to be a LR [16]. This fact can be formalized by extending the LR derivation for CPI (Appendix S1) to the CLR genotype listing using equation (7).
- LR1 and LR2 are calculated from computer-inferred genotypes using MLR, and so are LRs [48].

Since the log(LR) is a standard additive measure of information [50], [52], [53], it can be used to compare the relative information efficacies of all these DNA interpretation methods.

### Data

A set of STR data was generated to assess interpretation efficacy over a spectrum of two contributor mixture cases [21]. The experimental design has three axes: varying mixture ratios, serial DNA dilutions, and different contributor pairs. As shown in Table 1, the mixture weights were 10%, 30%, 50%, 70% and 90%. The DNA dilution amounts were 1 ng, 0.5 ng, 0.25 ng and 0.125 ng, standardized to a 25 µl PCR volume. DNA from two unrelated pairs of individuals was used to create two distinct sets of mixtures for a total of 40 (5 weights×4 amounts×2 pairs) mock sexual assault cases. After receiving these premixed DNA templates from the National Institute of Standards and Technology (NIST), we amplified them using a Promega PowerPlex16® STR kit under standard thermocycling conditions. We then detected the fluorescently labeled PCR products on an ABI 310® capillary sequencer to obtain size separated STR data signals.

**Table 1:** Forty DNA mixture samples were used in the study.

		10%	30%	50%	70%	90%
***A+G pair***	**1.0 ng**	B1	C1	D1	E1	F1
	**0.5 ng**	B2	C2	D2	E2	F2
	**0.25 ng**	B3	C3	D3	E3	F3
	**0.125 ng**	B4	C4	D4	E4	F4
***H+N pair***	**1.0 ng**	I1	J1	K1	L1	M1
	**0.5 ng**	I2	J2	K2	L2	M2
	**0.25 ng**	I3	J3	K3	L3	M3
	**0.125 ng**	I4	J4	K4	L4	M4

There are two contributor pairings, female A with male G, and female H with male N. The culprit mixture weights are 10%, 30%, 50%, 70% and 90%. The total DNA quantities used in a 25 µl PCR volume were 1.0 ng, 0.5 ng, 0.25 ng and 0.125 ng.

### Software

For quantitative genotyping, we applied Cybergenetics TrueAllele® Casework implementation of the described inference model to these STR data. After generating quality checked quantified peaks in the TrueAllele Analysis module, we then uploaded these data peaks to the TrueAllele database. We had TrueAllele quantitatively interpret the 40 mixture samples using a victim reference (as a one unknown contributor process), and also not using the reference (as a two unknown contributor process). Following quantitative TrueAllele inference, the genotype pmf 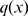 in each case was compared with the known suspect genotype 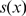 relative to a random genotype 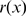 constructed from the alleles of 5,000 anonymous (actual, not simulated) individuals to form a log(LR) match score.

In the qualitative genotyping procedure, we applied a fixed threshold of 100 rfu to the peak data at each locus of a case experiment to generate its allele lists. For the CPI method, the computer formed a genotype list of all N allele pairs and associated a uniform probability 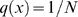 with each allele pair candidate x. For CLR, whenever there were no more than four peaks over threshold and the victim's alleles were included in those peaks, the computer filtered the candidate list using the victim reference genotype; otherwise, the initial “all allele pairs” list was used.

### Parameters

A match comparison Q = S at a locus between questioned genotype Q and suspect genotype S tests whether some Q genotype value equals some S genotype value. When such a match occurs, it has a positive probability value, i.e., 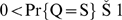. For example, in qualitative comparisons, this would mean an “inclusion” of suspect genotype S alleles in the data peak allele set. Alternatively, with no genotype value in common, event Q = S is not observed and there is a genotype mismatch, with 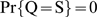. With a quantitative likelihood statistic, a zero match probability is not meaningful, e.g., it has an undefined logarithm.

Clearly, a match probability estimate based on real data can never actually equal zero – there is inherent scientific uncertainty that precludes making such a definite statement. Instead, we recognize that an STR experiment may produce uninformative data. We can use the probability of observing such data to impose a practical lower bound on the reported LR value. A log(LR) becomes negative when the probability of a specific match 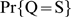 is less than that of a random match 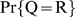. Assuming (fairly conservatively with ambiguous mixture or low template STR data) that uninformative data arises at least once in a thousand experiments (i.e., has a probability≥10^−3^), we set a lower bound of −3 on negative 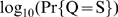 values. Also, to avoid potential confusion, the following data analyses only include case results having a positive total information value when all the (positive and negative) locus log(LR) values are added together.

## Results

We compared the described mixture interpretation methods on a DNA data set of varying mixtures and quantities, using log(LR) as a measure of DNA match information. We show that quantitative interpretation methods work much better than qualitative ones. We then analyze the effect of mixture weight on match information. Using linear regression on a log-log scatter plot, we explore the relative sensitivity of these different methods by examining the effect of DNA quantity on match information. Finally, we describe our experience in using these quantitative DNA interpretation efficacy comparisons in a courtroom setting.

### Match Information Comparison

We want to determine whether there is any appreciable difference between quantitative modeling and qualitative inclusion genotype inference methods. Since our task is DNA identification, we use as our metric the log(LR) match rarity statistic [54], which summarizes the relevant investigative and evidentiary identification information. The MLR assesses the identification information extracted from the data inferred by a genotype Q, as represented in its posterior pmf 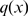. Different DNA mixture interpretation methods typically produce different genotypes and distributions.

**Figure 5:**
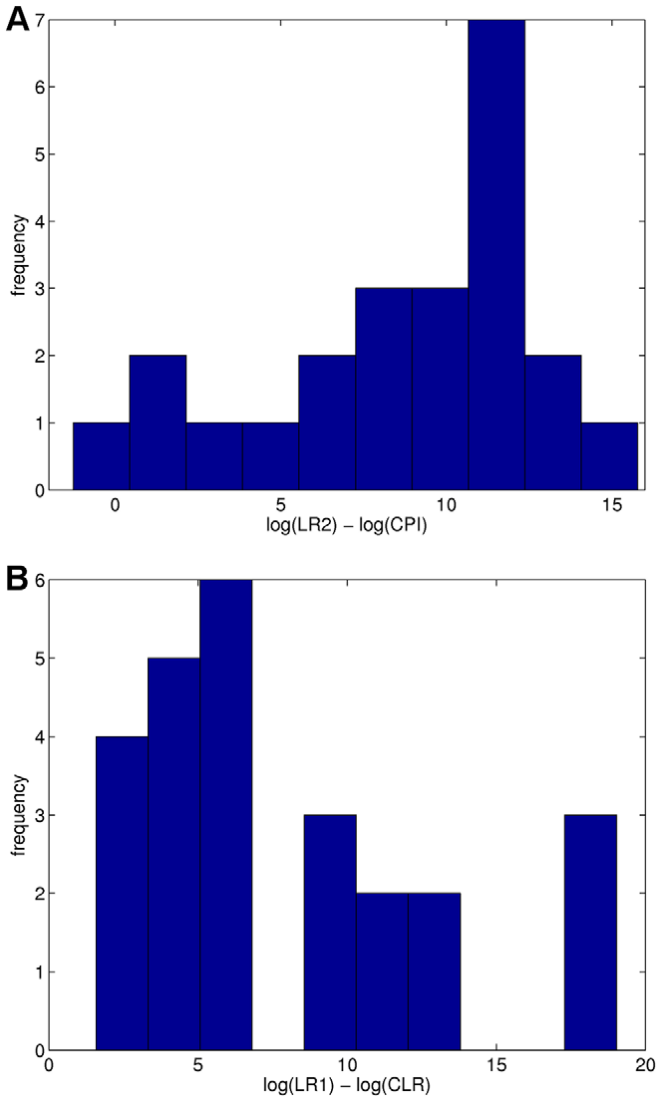
Histograms show how match information increases when using quantitative interpretation methods. The within-case log(LR) match information differences between quantitative and qualitative interpretation methods. (a) The information improvement log(LR2/CPI) between the LR2 (quantitative interpretation) and CPI (qualitative interpretation) match statistics on the same cases, when there are two unknown contributor genotypes. (b) The information improvement log(LR1/CLR) between the LR1 (quantitative method) and CLR (qualitative method) match statistics on the same cases, when the victim genotype is known, and there is one unknown contributor genotype.

When the victim genotype is not known during interpretation, we can compare the two-unknown quantitative modeling method LR2 with the qualitative CPI inclusion method (CPI threshold set at 100 rfu). Of the 40 cases (B1, B2, …, F4) processed using the two methods, LR2 produced a positive log(LR) in 39 of them, whereas CPI had a positive log(LR) in 23 cases. The ratio log(LR2/CPI) of these statistics for a given case equals the difference log(LR2)−log(CPI), which gives the order of magnitude improvement in match information of one method relative to the other. A histogram of these information differences for the 23 comparable cases is shown (Figure 5a), having a mean improvement of 8.57 log LR units and a standard deviation of 2.10. Thus we conclude that quantitative genotype inference is more informative than qualitative inference on this set of mixture cases having two unknown contributors.

With victim genotype A assumed to be known for mixture interpretation, we can compare the one-unknown quantitative modeling method LR1 with the qualitative CLR inclusion method (CLR threshold set at 100 rfu). Of the 40 cases (B1, B2, …, F4) with known profile A processed using the two methods, LR1 produced a positive log(LR) in all cases, whereas CLR had a positive log(LR) in 25 cases. A histogram of the match information differences log(LR1)−log(CLR) for these 25 comparable cases is shown (Figure 5b), having a mean increase of 7.90 log LR units and a standard deviation of 2.25. We therefore conclude that quantitative genotype inference is more informative than this qualitative approach on these mixture cases with one unknown contributor.

### Mixture Weight Effect

**Figure 6:**
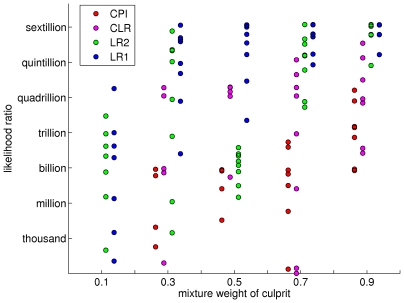
Match information as a function of mixture weight and interpretation method. A scatter plot of log(LR) match information (y-axis) versus culprit mixture weight (x-axis). Each of the five culprit weights (10%, 30%, 50%, 70% and 90%) has four columns, one for each of the mixture interpretation method match statistics (CPI in red, CLR in purple, LR2 in green, and LR1 in blue). In each column, there are up to eight mixture cases; a case is not shown when its log(LR) is negative.

To understand why quantitative genotype inference noticeably improves on qualitative inference, we examine the effect of mixture weight. For each case interpretation, we show its culprit mixture weight (x-axis) and log_10_(LR) match information (y-axis, in log_10_(LR) units) on a scatter plot (Figure 6). Each interpretation method is assigned its own color: for the quantitative methods, the one unknown LR1 as blue and the two unknown LR2 as green, and for the qualitative methods, the one unknown CLR (purple) and the two unknown CPI (red). Each of the five mixture weight columns shows scatter plots for all eight cases (four DNA quantities, two individual pairs) in each of four interpretation methods. Only positive log(LR) values are shown.

Beginning at the right (Figure 6) with the easiest major contributor cases having a 90% culprit mixture weight, we see that all four methods produced some positive result. The known victim LR1 quantitative method (blue) yielded the most information, closely followed by the two contributor LR2 modeling method (green). The qualitative inclusion methods extracted less information, with the known victim CLR method (purple) being more informative than the data-only CLR approach (red).

With a 70% major unknown contributor, the quantitative methods (blue, green) largely retain their information (Figure 6). Some of the qualitative known victim CLR cases (purple) remain informative, though others produce locus mismatches that reduce the log(LR) to near zero. The qualitative CPI method becomes far less informative than it was with a 90% culprit contributor.

A 50% mixture has equal contributions from both contributors, but with a known victim reference most information is retained (blue, purple). With two unknown contributors, there is a marked reduction in identification information (green, red). While unequal contributions can help identify which genotypes go with which unknown contributor, an equal 50% mixture weighting does not facilitate this identification, so (as with the qualitative approach) all the different genotype combinations become possible, thereby reducing information.

With a 30% minor culprit contributor, the quantitative methods (blue, green) retain considerable information. For most of these cases, the two unknown LR2 information (green) increases greatly relative to the 50% situation, reflecting the better separation of contributor genotypes. However, the qualitative methods do not perform as well with a minor contributor mixture – we see that about half of them no longer produce a positive log(LR). The remaining cases tend to have less information than in a 50% mixture.

At a 10% mixture weight, the quantitative methods still provide positive identification information (blue, green). One of the LR2 cases (green) has disappeared, but all the other LR1 and LR2 cases have positive log(LR). By comparison, there are no informative results for any of the CPI or CLR qualitative mixture methods, as indicated by the absence of red and purple cases.

### DNA Quantity Effect

**Figure 7:**
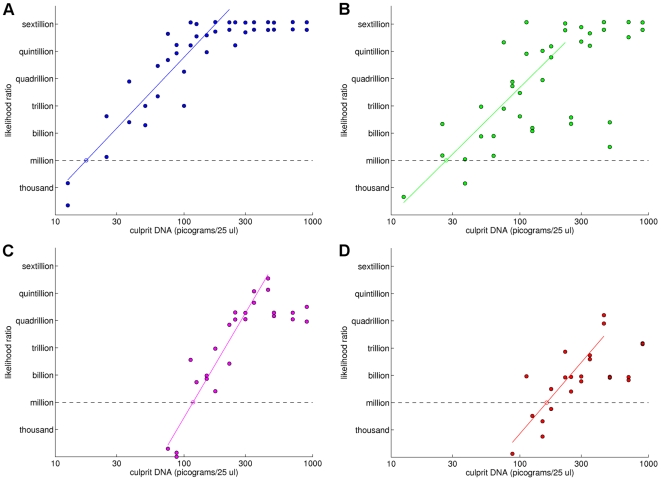
Determining DNA mass detection sensitivity by linear regression of match information versus DNA quantity. Scatter plots showing log(LR) match information (y-axis) versus log(culprit DNA) (x-axis) for four different mixture interpretation methods: (a) LR1 (blue), (b) LR2 (green), (c) CLR (purple), and (d) CPI (red). For each method, the scatter plots show an increasing ramp function that levels off when the maximum match information has been attained. The left ramp component is fitted to a regression line. The point at which this line intersects the horizontal million-to-one match information level gives the sensitivity of the interpretation method, measured in picograms of culprit DNA.

We can refine our initial observations about statistical difference and mixture weights by modeling the effect of DNA quantity on match information. The quantity of culprit DNA present in a mixture is the product of mixture weight and total DNA quantity. These properties are known from the study design for every case (Table 1), and so we know the culprit DNA quantity as well. It is instructive to examine a scatter plot that graphs LR information versus culprit DNA quantity for the cases. We present such plots on a log-log scale for all four interpretation methods (Figure 7).

Consider the quantitative LR1 method on the data set (Figure 7a, blue). For each of the 40 cases, a scatter plot shows the base ten logarithm of the quantity of culprit DNA (x-axis) and the match information log_10_(LR) (y-axis). Notice that in the 10 to 100 pg region (1 to 2 log mass units), the match case data linearly ramp up to the maximum match information value of 22. Beyond that point, in the 100 to 1000 pg region (2 to 3 log mass units), the information data remains constant at this maximum. The linear ramp along the left edge of the scatter plot shows how match information decreases with decreasing culprit DNA quantity. The linear regression line fits these case data with r^2^ = 0.83. This regression line intersects the million to one LR level at a log value of 1.24 (circle), or 17 pg. Thus the sensitivity of the LR1 method for detecting an evidentially useful DNA match on this data set is a culprit DNA quantity of 17 pg (Table 2, LR1).

**Table 2:** The mass detection sensitivity of different DNA mixture interpretation methods.

	Quantitative		Qualitative	
	LR1	LR2	CLR	CPI
**Sensitivity**	17 pg	27 pg	117 pg	162 pg
**95% Interval**	[9, 33]	[11, 64]	[82, 168]	[98, 266]

The sensitivity of each DNA mixture interpretation method on the data set was determined as the culprit DNA mass (in picograms) at which the log-log regression line intersected the million-to-one match information level. Confidence intervals for culprit mass DNA detection are shown for each method.

The quantitative LR2 method (without a victim reference) is shown (Figure 7b). We performed the same regression ramp analysis as above on the LR2 data points, producing a linear regression line (r^2^ = 0.63) that is shifted 0.19 log mass units to the right of the LR1 regression line. The LR2 line intersects the 10^6^ evidentiary threshold line at 27 pg. Not using the victim genotype reduces the sensitivity of quantitative genotyping, as measured by match information (Table 2, LR2).

We conducted this match information sensitivity analysis for both qualitative inclusion methods (Figures 7c and 7d). With abundant culprit DNA, the CLR method is seen to be more informative than CPI. At lower DNA template amounts, the culprit's allelic mixture peaks begin to fall below the preset threshold (e.g., 100 rfu). When the threshold discards true alleles, these qualitative methods can no longer infer a correct culprit genotype. The resulting locus mismatches then reduce the LR. On this data set, the CLR method (which uses the victim genotype) has a sensitivity of 117 pg, while the two unknown genotype CPI approach is seen to be the least informative, with a sensitivity of 162 pg (Table 2, CLR and CPI).

**Figure 8:**
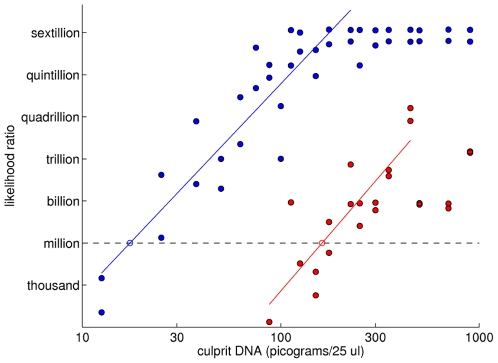
The information gap in detection sensitivity between quantitative and qualitative DNA interpretation methods. Two log-log scatter plots are shown of LR match information (y-axis) versus culprit DNA quantity. There is an order of magnitude information gap between the more sensitive quantitative LR1 interpretation method (blue) and the less informative qualitative CPI method (red).

There is an order of magnitude *information gap* between the quantitative and qualitative interpretation methods, evident from the scatter plot data, regression lines, and match sensitivities. Visually, the gap appears as a one logarithmic mass unit shift to the right of the qualitative methods (Figure 8). Whereas quantitative modeling has a match sensitivity limit in the ten picogram range, the less informative qualitative inclusion sensitivity is in the hundred picogram range (Table 2). The information difference between using quantitative data with a known victim genotype (Figure 8, blue; Table 2 LR1) and a qualitative review that ignores both quantitative data and the known genotype (Figure 8, red; Table 2 CPI) is a ten-fold sensitivity factor (17 pg vs. 162 pg) in the interpretable DNA quantity.

The actual sensitivity levels will vary between different data sets. Key factors in this variation include sample preparation, the extent of allele overlap between the genotypes contributing to the evidence, the suspect genotype, and allele population frequencies. However, the following case example further supports the observed ten-fold sensitivity improvement of quantitative over qualitative interpretation.

### Forensic Evidence

One of the authors recently had the opportunity to apply these DNA gap linear regression results in a criminal trial (Commonwealth of Pennsylvania v. Kevin J. Foley, Indiana County, No. 1170, Crim 2009). In April, 2006, dentist John Yelenic was slashed to death in his Blairsville home in Pennsylvania (PA). Although the murder weapon was never found, DNA was recovered from the victim's fingernails. The FBI laboratory's STR analysis produced quantitative data at the 13 CODIS loci, showing that most of the DNA came from the victim, and only 6.7% from an unknown contributor. The victim's estranged wife's boyfriend, PA State Trooper Kevin Foley, had a scratch on his forehead after the crime. The FBI compared their STR fingernail evidence data with suspect Foley's genotype, and reported a CPI match statistic (at 11 loci) of 13 thousand relative to a Caucasian population. With the key physical evidence well below the million to one persuasion level [51], the prosecution brought in Dr. Perlin as an outside DNA expert to provide a quantitative computer review of the data.

The outside expert ran the TrueAllele system on the two contributor mixture data, searching for one unknown genotype. (The victim was assumed to contribute to his own fingernail specimen; moreover, his genotype comprised 93.3% of the mixture.) The TrueAllele interpretation yielded a genotype Q, along with a pmf 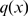, at 12 STR loci; the TPOX locus was not used because of bleed-through artifact. The MLR of genotype Q with defendant Foley's genotype S (relative to the population genotype R) was 189 billion, much greater than the jury “convincing threshold” of one million. The defense lawyers sharply questioned the computer's statistically inferred LR, given its seven order of magnitude improvement over the FBI's CPI result. The prosecution presented the DNA information gap (Figure 8) to show that the observed match score disparity was entirely expected, as follows.

**Figure 9:**
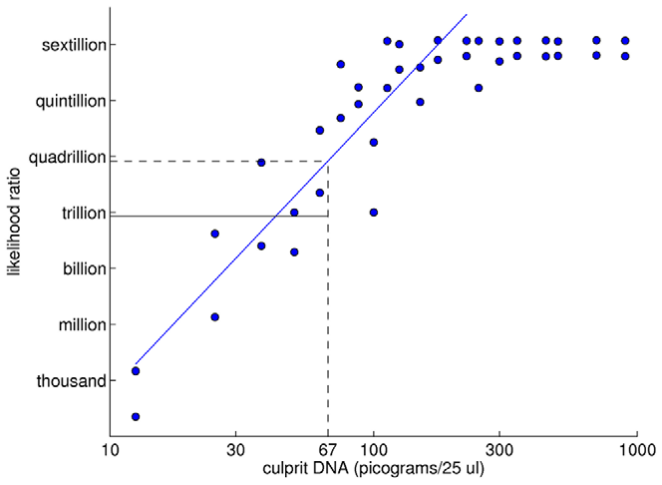
Using a linear relationship to predict match information from DNA quantity in a criminal case. The linear regression of log(LR) match information (y-axis) versus log(culprit DNA) (x-axis) is used here as a calibration curve for the LR1 method. For a mass of 67 pg of culprit DNA, the regression line shows an expected LR of 10^15^. With the 12 STR loci actually examined in the homicide case (instead of the validation study's 15 STR loci), we expect an 80% information reduction to a LR of approximately 10^12^.

The expert explained the linear regression in the log-log LR1 validation study scatter plot to the court, using the regression line as a calibration curve (Figure 9). The TrueAllele computer had determined that the unknown culprit comprised 6.7% of the mixed specimen; the FBI laboratory measured the starting DNA quantity as 1 ng (in a standard 25 µL volume). Therefore, the unknown culprit DNA quantity was 67 pg (i.e., 6.7% of 1,000 pg). Locating 67 pg on the x-axis, we see that it intersects the calibration line at a y-axis value of 14.75 log_10_(LR) information units. Since our analysis used only 12 of the validation study's 15 STR loci, we take 80% of this additive 14.75 information value to find an expected log_10_(LR) result of 11.80. The standard deviation about the regression line is 2.28, so (accounting for the 80% shrinkage) the 95% confidence interval at 67 pg is [8.15, 15.45]. The observed match information of 11.28 (or, log_10_(189×10^9^)) is centered within the confidence interval, near the predicted 11.80 regression value.

The validation data in this paper therefore do not support the defense hypothesis that the quantitatively inferred LR of 189 billion is unexpectedly high. Nor is the FBI's reported CPI match statistic of 13 thousand unexpectedly low. As the FBI has reported, their qualitative threshold-based CPI method does not extract much match information from LT-DNA mixture evidence [18]. This is understandable – CPI ignores the victim genotype, and discards quantitative peak height data. It is generally accepted in the forensic inference and statistics community that the more informative “LR approach is preferred” since it “makes full use of the evidence” in these low DNA situations [16]; our DNA information gap (Figure 8) results support this broad consensus view. After hearing testimony on both (the qualitative and quantitative) DNA match statistics, the jury convicted former Trooper Foley of first-degree murder.

## Discussion

DNA evidence can be used to establish a match between biological specimens. However, the match result and its statistical rarity may depend on how this evidence is interpreted. In the STR process, both the essential PCR amplification and DNA sequencer readout steps are linear operations, combining materials additively to produce a total quantitative signal. This additive feature can be modeled mathematically, enabling prediction of the observed quantitative data pattern from underlying statistical parameters. Using modern computational methods, this linear model can be used to quantitatively interpret the data, inferring underlying genotypes as probability distributions that can then be matched against other genotypes.

Although some American laboratories are moving to quantitative modeling of DNA mixture data, most still use CPI [13], [55]. Their analysts apply thresholds to data peaks to decide whether or not they believe that an evidence peak represents an allele in the genetic material. With large quantities of culprit DNA, this determination can be reliable. In such cases, the analyst assembles lists of alleles, translates them into lists of genotypes, and afterwards makes match comparisons to suspect genotypes. However, more complex data that has mixtures or LT-DNA limits the applicability of such qualitative procedures [13], [18].

The two person mixture sensitivity results presented here show that the match information depends on both the DNA quantity of the unknown contributor and on the interpretation method. Below a million-to-one LR, jurors begin to question the persuasiveness of DNA evidence. Quantitative linear models that use peak height patterns can form data-adapted genotype probability distributions that provide the requisite 10^6^ match information with a sensitivity level of around 10 pg. However, qualitative threshold-based methods produce flat genotype distributions that are less informative, with a sensitivity level of around 100 pg. The less sensitive qualitative approach may therefore be less relevant below this DNA mass level.

Crime laboratories interpret DNA evidence within the sensitivity limits of their interpretation methods. When using qualitative threshold-based methods, they seek criteria for processing only those samples having enough DNA to elevate data peaks above threshold [18], [56]. Another strategy is to report only genotype matches for major contributors to a mixture [12]. Given the 100 pg match sensitivity limit for qualitative interpretation (Table 2), it makes sense for them to not expend considerable effort qualitatively analyzing DNA evidence that has low amounts of culprit DNA.

A more proactive solution is to recognize the inherent *information gap* between the match sensitivity of older qualitative and newer quantitative interpretation methods. While the 10 pg to 100 pg DNA sensitivity range may be impervious to qualitative review, these low template data are amenable to more informative quantitative interpretation. Moreover, these resolvable DNA levels commonly occur in property crime, as well as in “difficult” sexual assault and homicide evidence. Informative genotypes and matches can help law enforcement make DNA identifications of dangerous criminals. Crime laboratories need not ignore or discard LT-DNA evidence. Instead, society can have them employ appropriately informative DNA interpretation methods for resolving and preventing crime.

## Supporting Information

Appendix S1 Probability of inclusion is a likelihood ratio (proof).
(0.37 MB PDF)
