# Supplementary material for: An Information Gap in DNA Evidence Interpretation
Source: PLoS One. 2009 Dec 16;4(12):e8327. doi: 10.1371/journal.pone.0008327 (PMC2791197; doi:10.1371/journal.pone.0008327)
Supplement: Appendix S1 — Probability of inclusion is a likelihood ratio (proof). (0.37 MB PDF) [file pone.0008327.s001.pdf]

## Appendix: Probability of Inclusion is a Likelihood Ratio

The probability of inclusion (PI) is traditionally viewed as a DNA match statistic quite distinct from a LR [13,16]. This is because PI makes a statement about allele sets, rather than genotypes, making it difficult to formulate a hypothesis for a LR about genotype assumptions. However, the standard data LR can be expressed as an equivalent match LR in terms of genotypes and their pmfs [48]. Within this match LR framework, we show that PI is, in fact, a LR.

### *Constructing Genotypes*

We begin by constructing a canonical genotype *Q* that PI implicitly forms from a set of alleles *I*. This allele inclusion set *I* is obtained from STR locus data by retaining *K* alleles whose peak heights exceed some predetermined threshold. The pmf of genotype *Q* is a probability distribution completely characterized by a prior probability and a likelihood function [57]. The hallmark of PI is its symmetry in treating alleles, which is the source of both its simplicity and its loss of information. A natural prior $\pi(x)$ that expresses this symmetry is the uniform distribution on genotypes.

We can form an inclusion likelihood function $\lambda(x)$ for the inclusion method. The only included allele pairs $x$ are those for which both alleles are included in allele inclusion set $I$, so we define a genotype value inclusion subset $G$ of $X$ as

$$G = \{[i\ j] \mid i, j \in I, i \leq j\}$$

that contains just these allele pairs. From the symmetry of PI, we then define its genotype likelihood function $\lambda(x)$ as the indicator function on support set $G$, or

$$\begin{aligned} \lambda(x) &= \chi_G(x) \\ &= \begin{cases} 1, & x \in G \\ 0, & \text{otherwise} \end{cases} \end{aligned}$$

From Bayes theorem, the posterior probability distribution *q(x)* for PI is the product of *π(x)* and likelihood *λ(x)* as

$$q(x) \propto \pi(x) \cdot \lambda(x)$$

With $K$ alleles in $I$, there are $N = K(K+1)/2$ distinguishable allele pairs in genotype support set $G$. After normalizing so that the sum of the pmf probability values equals one, we obtain the PI pmf *q(x)* as

$$q(x) = \begin{cases} \frac{1}{N}, & x \in G \\ 0, & \text{otherwise} \end{cases}$$

Now suppose that there is a suspect with genotype $S$ having a unique allele pair $g$ that is included in the PI genotype set $G$. The pmf *s(x)* is

$$s(x) = \begin{cases} 1, & x = g \\ 0, & \text{otherwise} \end{cases}$$

To form a LR, we specify a reference population having genotype $R$ and pmf *r(x)*, where

$$r(x) = \begin{cases} p_i^2, & \text{homozygote x} \\ 2p_i p_j, & \text{heterozygote x} \end{cases}$$

Here, $p_i$ is the frequency in the population of allele $i$.

***The Match Statistic***

The PI match statistic is defined by the allele frequencies of included alleles as [2]

$$\text{PI} = \frac{1}{\left(\sum_{i \in I} p_i\right)^2}$$

Expanding the sum of products in the denominator gives

$$= \frac{1}{\sum_{i \in I} p_i^2 + \sum_{i,j \in I, i \le j} 2p_i p_j}$$

The first summation describes the population frequencies of all the included homozygote genotypes, while the second describes the included heterozygote genotype frequencies. We can combine these using the pmf *r(x)* of population genotype *R* as

$$= \frac{1}{\sum_{x \in G} r(x)}$$

restricted to the inclusion genotype set *G*.

Since matching allele pair *g* is in genotype inclusion set *G*, $\sum_{x \in G} s(x) = 1$. So we can write

$$\text{PI} = \frac{\sum_{x \in G} s(x)}{\sum_{x \in G} r(x)}$$

Multiplying numerator and denominator by $1/N$, and distributing this constant value into the sums, we then have

$$= \frac{\sum_{x \in G} \frac{1}{N} \cdot s(x)}{\sum_{x \in G} \frac{1}{N} \cdot r(x)}$$

But this uniform weighting of $1/N$ on genotype support set *G* is just the evidence pmf *q(x)*, and so

$$= \frac{\sum_{x \in X} q(x) \cdot s(x)}{\sum_{x \in X} q(x) \cdot r(x)}$$

where the summation now ranges over the entire set *X* of possible genotype values.

The probability of genotype equality $\Pr\{Q = S\}$ can be written [48] as a summation of genotype pmf products $\sum_{x \in X} q(x) \cdot s(x)$. Similarly [48], $\Pr\{Q = R\} = \sum_{x \in X} q(x) \cdot r(x)$. Therefore, PI can be written as

$$\text{PI} = \frac{\Pr\{Q = S\}}{\Pr\{Q = R\}}$$

This ratio of genotype match probabilities is a likelihood ratio for the standard inclusion hypothesis that the suspect's DNA is in the evidence [48]. Therefore, PI is a LR.

We have demonstrated that by constructing a canonical inclusion evidence genotype $Q$, the usual PI statistic can be viewed as a LR. This means that the strengths and weaknesses of the inclusion method can be understood through its genotype $Q$ and uniform pmf $q(x)$. Moreover, treating PI as a LR enables us to examine its information content log(LR), and make meaningful comparisons with other LR results.
